# Response to: ‘The diagnosis of diabetic striatopathy should only be made after careful exclusion of all possible differential diagnoses’

**DOI:** 10.51866/lte.26-00056

**Published:** 2026-09-29

**Authors:** Say Yee Loo, Nadiah Abdul Rahim, Hana Azhari, Ezura Madiana Md Monoto, Saharuddin Ahmad

**Affiliations:** 1 Department of Family Medicine, Faculty of Medicine, Universiti Kebangsaan Malaysia, Jalan Yaacob Latif, Bandar Tun Razak, Cheras, Kuala Lumpur, Malaysia.; 2 Klinik Primer HCTM,UKM, Department of Family Medicine, Jalan Dwitasik, Bandar Sri Permaisuri, 56000 Cheras, Kuala Lumpur, Malaysia.

**Keywords:** Chorea, Type 2 diabetes mellitus

**Dear Editor,**

We thank the authors for their interest in our published case report entitled ‘Diabetic striatopathy: A case report of haemichorea in an older adult woman with poorly controlled diabetes’.^1,2^ We appreciate the important points raised in their letter and welcome the opportunity to clarify the diagnostic considerations and provide additional information to the readers.

**Differential diagnosis of diabetic striatopathy and chorea (Points 1 and 2)**

We appreciate the authors’ emphasis on the various causes of chorea and agree that diabetic striatopathy should only be diagnosed after careful consideration and exclusion of other possible causes.

The absence of infective symptoms including fever, diarrhoea, rigors, headache, vomiting, generalised weakness or changes in mental status,^3^ as well as negative infective screening (Table 1), and the absence of high-risk behaviours made infective causes unlikely. Additionally, during the initial assessment and subsequent reviews, the patient did not demonstrate bradykinesia, tremor, rigidity or postural instability that would suggest Parkinson’s disease.^3^ We also ruled out acute cerebrovascular accidents and space-occupying lesions through a computed tomography scan of the brain.

**Table 1:** Summary of the patient’s blood parameters.

Blood test (upon presentation)	Result	Normal range	Unit
Random blood sugar level	14.2	<7.8	mmol/L
HbAlc level	10.3	<5.7	%
Lactate level	1.9	0.6-1.4	mmol/L
Urine ketone	Negative		
Haemoglobin level	10.0	12.0-15.0	g/dL
Platelet count	220	150-410	x10^9/L
Total white blood cell count	8.2	4.0-10.0	x10^9/L
Urea level	12.8	2.5-6.7	mmol/L
Sodium level	138	136-145	mmol/L
Potassium level	5.1	3.5-5.1	mmol/L
Creatinine level	184	49-90	mmol/L
Calcium level	2.39	2.2-2.60	mmol/L
Magnesium level	0.75	0.66-1.07	mmol/L
Phosphate level	1.33	0.81-1.45	mmol/L
**Venous blood gas**	Result		
Venous pH	7.35	7.35-7.45	
Bicarbonate level	22.7	22.0-28.0	mmol/L
Glucose level	14.0	3.5-5.4	mmol/L
Lactate level	1.9	0.0-2.0	mmol/L
Estimated serum osmolality (calculated based on 2[Na]+glucose)^4^	290		mOsm/kg
**Blood test (5 years ago)**			
ANAIF	Negative		
Rheumatoid factor	Negative		
Extractable nuclear antibodies	Negative		
HIV Ag/Ab	Non-reactive		
AntiHCV	Non-reactive		
HbsAg	Non-reactive		
AntiHBS	Reactive (136.5)		mIU/mL

HbAlc: Haemoglobin A1c; ANAIF: antinuclear antibody by indirect immunofluorescence; HIV: human immunodeficiency virus; AntiHCV: hepatitis C virus antibody; HbsAg: hepatitis B surface antigen; AntiHBS: hepatitis B surface antibody

Furthermore, the late onset, postmenopausal status, acute presentation and focal right-sided symptoms made hereditary causes less likely. Genetic testing was not performed because of the patient’s late age of onset and the absence of a family history suggestive of a hereditary or genetic disorder. Given her negative autoimmune screening (Table 1) and the absence of family history, an autoimmune cause was also considered less likely.

Based on the clinical presentation, investigation findings and exclusion of other relevant differential diagnoses, diabetic striatopathy was considered the most likely diagnosis.

**Magnetic resonance imaging (MRI), electroencephalogram (EEG) and cerebrospinal fluid (CSF) testing (Points 3–5)**

We acknowledge that MRI, EEG and CSF analysis are important in excluding other potential causes. However, access to these investigations in our primary care setting is limited. The case was appropriately referred to the neurology team. Risperidone was initiated, and her glycaemic control was optimised with improvement in the abnormal involuntary movements. Unfortunately, the patient was subsequently admitted twice for new onset of decompensated heart failure. Her subsequent clinical deterioration limited the feasibility of continuing her further neurological investigations.

**Temporal course of illness (Point 6)**

We understand the concern that osmotic demyelination following rapid correction of hyperglycaemia may have contributed to the chorea. However, this was considered less likely in our patient. The abnormal involuntary movements had begun about 3 weeks before the presentation during a period of poor glycaemic control following discontinuation of certain oral hypoglycaemic agents due to deteriorating renal function.

**Table 2:** Timeline of illness

Timeline	Issue	Investigation	Management
2 months prior	Adjustment of OHA due to worsening of renal function	HbAlc level: 6.7% (4 months prior) eGFR from 42 to 23 mL/min/1.73 m^2^	**Discontinued:** Metformin and gliclazide (for renal dosage adjustment) **Initiated:** Empagliflozin 10 mg OD (for optimisation of glycaemic control and renal protection)
1 month prior	Intolerance to empagliflozin Persistent poor glycaemic control	SMBG level: 16–28 mmol/L	**Discontinued:** Empagliflozin **Restarted:** Gliclazide MR 90 mg OD (the patient insisted on OHA only; refused insulin) Referral to a tertiary hospital was not indicated, as the patient did not have evidence of diabetic emergencies.
Day of presentation	Chorea occurred 3 weeks prior to presentation during a period of poor glycaemic control	Finger prick test result on arrival: 14.2 mmol/L Brain CT result: Subtle hyperdensity in the left striatum HbA1c level: 10.3%	Referral to the emergency department A family member persistently refused insulin and admission and insisted on optimisation of OHA despite poor glycaemic control. **Discontinued:** Gliclazide **Initiated:** Glimepiride 1 mg OD, sitagliptin 25 mg OD and risperidone 2 mg OD/BD
2 weeks after chore	Improvement in glycaemic control Resolving abnormal involuntary movements	SMBG level: 6.4–11.2 mmol/L	**Changed:** Sitagliptin 25 mg OD to linagliptin 5 mg OD

OHA: oral hypoglycaemic agent, SMBG: self-monitoring blood glucose, OD: once daily.

**Discrepancy with the statement of muscle tone (Point 7)**

We thank the authors for highlighting this error. We clarify that ‘dystonia’ was inaccurately included in the neurological examination findings. The patient’s muscle tone was normal during examination. The cerebellar examination findings were also normal.

**Patient’s regular medications (Point 8)**

We appreciate the authors’ comment regarding medication-related chorea, including dopamine- blocking/depleting agents, dopaminergic medications, antiseizure medications, central nervous system stimulants and calcium channel blockers except for amlodipine.^3^ Her regular medications were carefully reviewed as part of the assessment.

- Perindopril 8 mg once daily
- Spironolactone 25 mg once daily
- Atenolol 100 mg once daily
- Amlodipine 10 mg once daily
- Atorvastatin 20 mg once daily
- Gliclazide MR 120 mg once daily
- Vitamin B1 100 mg, vitamin B6 200 mg and vitamin B12 200 pg (Neurobion), one tablet once daily
- LA aqueous cream with glycerin 25%
- Extended-release metformin 2 g once daily — discontinued due to the need for renal dose adjustment
- Empagliflozin 10 mg once daily — discontinued due to intolerance

**Summary of the blood parameters with an added reference range (Point 9)**

We appreciate the constructive feedback about the need to include reference ranges to ease the readers’ interpretation of the investigation findings. We have included the reference ranges, selected venous blood gas results taken during her initial presentation and the results of her previous autoimmune and infective screening.

The osmotic symptoms mentioned in the article refer to polyuria and polydipsia, which may occur in severe hyperglycaemia.^5^

## Conclusion

We hope that our responses have adequately addressed the concerns raised and clarified the clinical reasoning in this case. We acknowledge the limitations in the investigations performed in view of the resource constraints within our setting, as well as the patient’s later clinical deterioration and recurrent hospital admissions and the family’s decisions regarding additional management. Nonetheless, the diagnosis was made based on the overall clinical presentation, available investigation findings and careful consideration of the relevant differential diagnoses. We hope that these additional details will provide the readers with a clearer understanding of the diagnostic and management challenges encountered in this case.

## References

[1] Abdul Rahim N, Loo SY, Ahmad S, Md Monoto EM, Azhari H (2026). Diabetic striatopathy: A case report of haemichorea in an older adult woman with poorly controlled diabetes. Malays Fam Physician..

[2] Mehri S, Finsterer J (2026). The diagnosis of diabetic striatopathy should be made only after careful exclusion of all possible differential diagnoses. Malays Fam Physician..

[3] Manon B, Oksana S (2026). Overview of Chorea.. Up To Date.

[4] Malaysian Endocrine & Metabolic Society (2020). Practical Guide to Inpatient Glycaemic Care..

[5] Dhatariya K, Mustafa O, Stathi D (2025). Hyperglycemic Crises. Endotext.

